# Multiparametric-MRI and Targeted Biopsies in the Management of Prostate Cancer Patients on Active Surveillance

**DOI:** 10.3389/fonc.2015.00004

**Published:** 2015-01-26

**Authors:** Kiri Sandler, Mausam Patel, Charles Lynne, Dipen J. Parekh, Sanoj Punnen, Merce Jorda, Javier Casillas, Alan Pollack, Radka Stoyanova

**Affiliations:** ^1^Department of Radiation Oncology, University of Miami, Miami, FL, USA; ^2^Department of Urology, University of Miami, Miami, FL, USA; ^3^Department of Pathology, University of Miami, Miami, FL, USA; ^4^Department of Radiology, University of Miami, Miami, FL, USA

**Keywords:** active surveillance, multiparametric-MRI, prostate cancer, radiotherapy, targeted biopsies

## Abstract

An important key to clinical management of prostate cancer patients is to determine early those who will benefit from primary treatment and are not good candidates for active surveillance (AS). We describe a 67-year-old gentleman with a long history of stable prostate-specific antigen (PSA) levels and a negative biopsy. After slight PSA rise and low volume Gleason score 6 biopsy, the patient was considered for primary treatment or AS. A multiparametric (MP)-MRI exam revealed a suspicious lesion in the anterior apex of the prostate. Biopsies were carried out on a 3D-ultrasound prostate biopsy system with MRI-fusion. The location of the target area was challenging and could have been missed using standard 12-core biopsy template. The pathology determined Gleason 3 + 4 disease in 30% of the core from this region. Consequently, the patient underwent radiotherapy (RT). MP-MRI was also used to follow the changes from pre- to post-RT.

## Introduction

Prostate cancer is multifocal and heterogeneous, with foci of different Gleason scores (GS) commonly present. The treatment decision-making process among active surveillance (AS), radiotherapy (RT), and surgery can be a difficult one and is primarily based on clinical–pathological data such as prostate-specific antigen (PSA), digital rectal exam (DRE), and prostate biopsy information, the latter of which is compromised by such heterogeneity. The clinically accepted method for the diagnosis of prostate cancer is transrectal ultrasound (TRUS)-guided biopsies following a 12-core template. TRUS does not reliably visualize cancer foci, with up to 30% of tumors being isoechoic (1) and a roughly 50:50 chance of documenting cancer in hypoechoic lesions. Utilizing multi-core template is still relatively a tumor-undirected approach and has the potential to miss high grade and/or anterior lesions that may dictate outcome.

## Background

**Figure 1:**
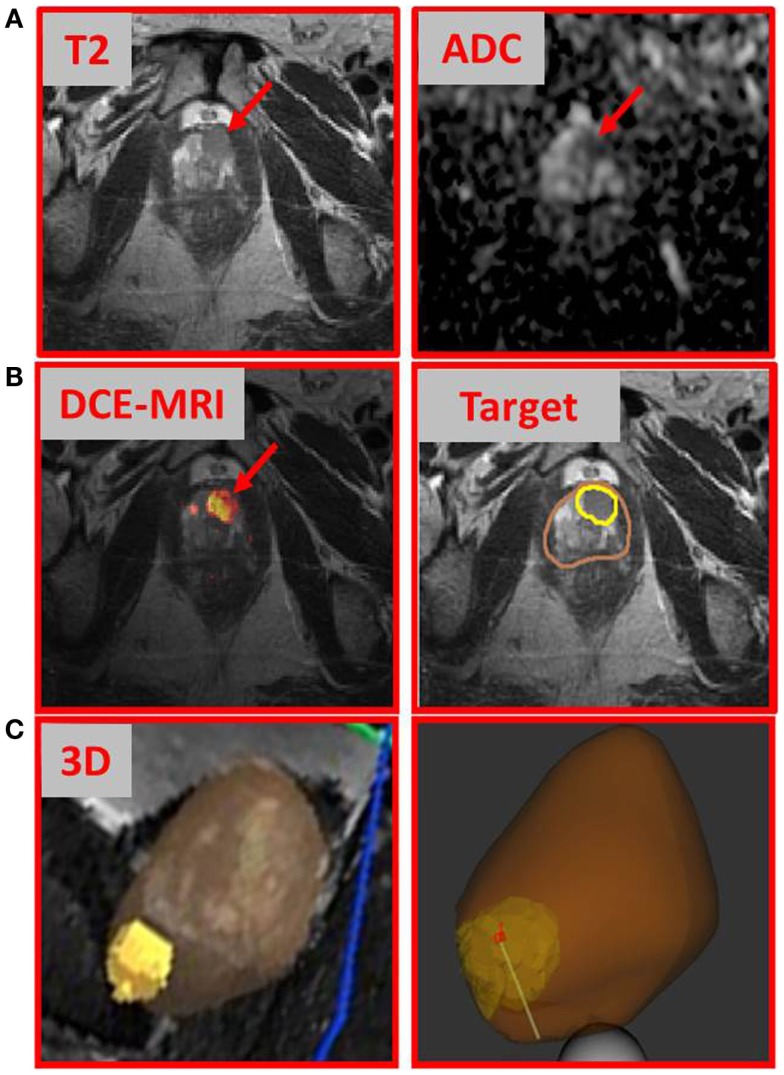
**Multiparametric-MRI findings and directed prostate biopsy of the index lesion**. **(A)** T2-weighted MRI and ADC map, with arrows indicating tumor area; **(B)** DCE-MRI intensity map with the lightest (yellow) part representing the areas of rapid contrast wash-in and gradual washout; and **(C)** tumor (yellow) and prostate (brown) contours. Both volumes were transferred to Artemis™ for fusion to the real-time ultrasound prior to prostate biopsy; 3D representation of the tumor with biopsy needle path shown.

We describe a 67-year-old gentleman who presented for evaluation of recently diagnosed cT1N0M0 prostate cancer. After many stable PSAs in the low 4 ng/ml range over 6 years, PSA rose slightly to 4.7 ng/ml a year ago. TRUS biopsy at this time was negative for malignancy, but showed a focus of high grade prostatic intraepithelial neoplasia in the left lateral base. Seven months later, the PSA rose to 6.2 ng/ml and a repeat biopsy was performed, which showed Gleason 3 + 3 = 6 prostatic adenocarcinoma involving two cores with <10% involvement from the left lateral base and left apex. TRUS-estimated prostate volume was 65 cc with a PSA density 0.095 ng/ml. Review of systems was positive for mild daytime urinary frequency and nocturia one to two times per night. DRE revealed a large, smooth prostate with no discrete abnormality. At this time, he was a candidate for either AS or primary treatment. Three months later (PSA = 7.2 ng/ml), the patient underwent multiparametric (MP)-MRI exam. MP-MRI, consisting of T2-weighted, T1 dynamic contrast-enhanced MRI (DCE-MRI), and diffusion-weighted MRI (DWI) with derived apparent diffusion coefficient (ADC) revealed a suspicious lesion in the anterior apex of the prostate. MP-MRI was acquired on a GE Discovery MR750/3 T using standard clinical protocols. Figure 1A shows an area of nodular hypointensity on the axial T2-weighted image marked with an arrow. The same area is associated with low ADC values (mean ± SD: 842 ± 67 μm^2^/s) relative to the surrounding peripheral zone tissue (mean ± SD: 1858 ± 168μm^2^/s; *p*-value < 0.001, Student’s *t*-test). DCE-MRI was analyzed with in-house developed software (2) and the area indicated in the heat map in Figure 1 was associated with fast contrast uptake and gradual washout. The gadolinium contrast pharmacokinetic parameters for this area: *K*^trans^ [volume transfer constant between plasma and extravascular space (EES)] and *k*_ep_ (rate constant between EES and plasma) are 0.351 and 0.705 min^−1^, respectively (3). In contrast, the same constants for the surrounding peripheral zone tissue were markedly lower: *K*^trans^ = 0.175 min^−1^ and *k*_ep_ = 0.292 min^−1^. Based on these findings, in addition to a PSA of 7.2 ng/ml, the patient underwent MRI-ultrasound-directed prostate biopsies.

We used a commercially available 3D-ultrasound (US) prostate biopsy system that allows for MRI-fusion at the time of the procedure (Artemis system, Eigen, Sun Valley, CA, USA). This is accomplished by (i) utilizing software to contour the prostate and biopsy target(s) (Figure 1B, “Target”); (ii) loading outlined MRI volumes into the system for fusion; (iii) using a TRUS end-fire probe to generate a 3D prostate image by rotating the probe cradle; and (iv) registration of the triangulated gland surfaces from both modalities using an adaptive-focus deformable model (4). The system tracking computes the needle trajectory, its core position, and its depth (Figure 1C). After biopsy acquisition, the tip of the biopsy is marked with ink for accurate localization of the tissue sample.

The biopsies were taken from a suspicious region not seen on TRUS but identified by MP-MRI in the apex. It should be noted that obtaining biopsies of this area is sometimes challenging and may not be sampled using a typical 12-core template.

## Discussion

**Figure 2:**
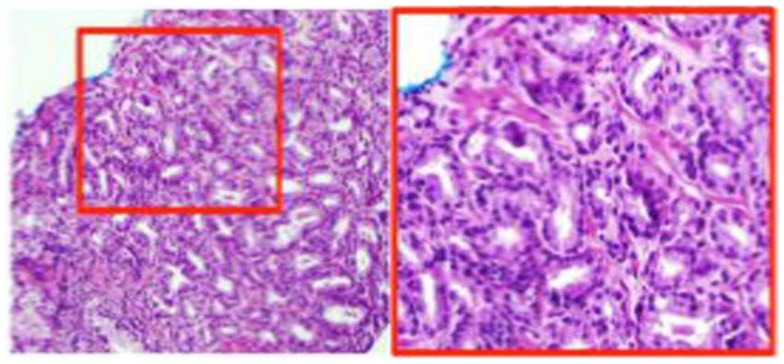
**H&E stain of MP-MRI-ultrasound-directed prostate biopsy**. The ink stain indicates that the tumor is located on the tip of the biopsy. The region in the red box is at 20× magnification and shows Gleason score = 3 + 4.

**Figure 3:**
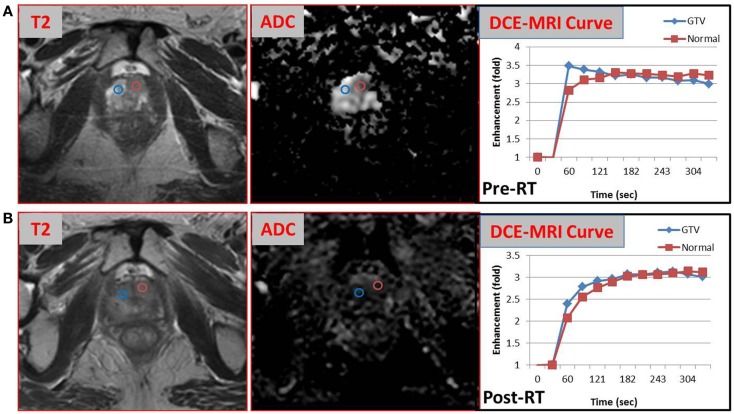
**Comparison of MP-MRI exam before and after radiotherapy**. T2-weighted MRI, ADC map, and contrast-versus-time curves pre- **(A)** and 3 months post-RT **(B)** of two prostate regions are shown: malignant lesion pre-treatment (blue) and healthy appearing PZ (red).

The pathology review determined GS 3 + 4 in 30% of the core from the suspicious region (Figure 2). Thus, the patient was not considered as a candidate for AS and he underwent RT. The patient was enrolled in an institutional investigator-initiated RT trial in which higher doses of radiation are delivered to the MP-MRI identified dominant lesion in half of the patients. He was randomized to the standard arm and the entire prostate and proximal seminal vesicles received 80 Gy in 40 fractions. Repeat MP-MRIs were performed 3 months post-RT. In Figure 3, the T2-weighted MRI, ADC map, and contrast-versus-time curves are shown pre- **(A)** and 3 months post-treatment **(B)** in two prostate regions. The comparison illustrates the disappearance of the lesion on the post-treatment T2 and ADC images. Post-treatment ADC values for the lesion (blue) were 1576 ± 80 μm^2^/s and for normal prostate (red) were 1467 ± 146 μm^2^/s. While quite distinct pre-treatment, the contrast-versus-time curves post-RT were similar with a temporal pattern characteristic for normal prostate.

## Concluding Remarks

Even with 12-core template grid-like sampling of the prostate, TRUS-guided biopsies have been reported to miss 30% of cancers at the time of first biopsy (5), and studies have shown limited accuracy in tumor localization when correlated with pathologic specimens (6).

T2-weighted MRI provides an excellent depiction of prostate anatomy with regions of healthy peripheral zone prostate tissue demonstrating higher signal intensity than prostate cancer. DWI is an imaging technique, which is sensitive to diffusion of water molecules and provides details about tissue architecture. The derived ADC values are significantly lower in tumor than in normal prostate. The lower the ADC value, the greater the chance of diagnosing GS ≥7 disease (7). DCE-MRI has also demonstrated that greater and earlier enhancement is seen in tumor versus normal tissue. DCE-MRI is a measure of tissue vascularity and hence angiogenesis. MP-MRI has been shown to improve the sensitivity and specificity of tumor localization (8). Combining these methods, results in better correlation with pathologic findings, with accuracy of up to 85% (9).

Death due to prostate cancer occurs late, but is significantly greater in men observed versus those treated primarily. An important key to clinical management is to determine early those who are not good candidates for AS. Recent reports show a 25–35% rate of upgrade in prostatectomy specimens in patients considered candidates for AS (10). The reported rates of conversion depend on the length of follow-up. For instance, these rates range from 20 to 33% in a review of several AS clinical trials, with median follow-up time from 1.3 to 3.5 years (11). In a single institution study with the longest available follow-up, Klotz et al. reported rates of conversion of 24.3, 36.5, and 45% at 5, 10, and 15 years follow-up, respectively (12). Patient anxiety and quality-of-life considerations may lead to conversion to treatment in the absence of biological progression (13). Klotz et al. reported that 6% of patients discontinued AS due to personal preference rather than clinical progression (12). Therefore, in men undergoing AS, we estimate a 20–30% rate of conversion to treatment by 3 years, suggesting that men in whom early conversion to treatment is recommended are probably those who have been undergraded and/or understaged by conventional assessments. Identifying early, those patients who will benefit from primary treatment and are not good candidates for AS, is of paramount importance. MRI/US-fused biopsy offers a more accurate way to target determinant lesions that might have been missed. With the trend toward more conservative management of prostate cancer, it is crucial to be able to identify such lesions early, to develop strategies that require fewer biopsies at each session, and to apply imaging techniques to better follow determinant lesions for progression and biopsy.

The advantages over standard ultrasound-guided biopsies are not only illustrated in the case presented but have been suggested by other studies. In a meta-analysis of 16 studies, Schoots et al. found that MRI-guided biopsy had a 20% better rate of detecting significant prostate cancer as compared to TRUS-guided biopsy (14). Moreover, MRI-guided biopsy was almost twice as likely to avoid detecting insignificant prostate cancer as compared to TRUS-guided biopsy. Thus, MRI guided biopsy is a promising procedure for avoiding the oversampling and overtreatment of insignificant disease. One can envision a lower frequency of biopsies and fewer directed biopsies. MP-MRI is becoming widely accepted and used in the selection and management of patients for AS. Given the emergence of technology that allows for fusion between MP-MRI and real-time ultrasound, clinicians can now direct biopsies toward regions most suspicious for harboring significant cancer based on MP-MRI. This opens the door for a paradigm shift in how patients are selected and managed for AS.

## Conflict of Interest Statement

The authors declare that the research was conducted in the absence of any commercial or financial relationships that could be construed as a potential conflict of interest.
